# Gut epithelial barrier dysfunction in lupus triggers a differential humoral response against gut commensals

**DOI:** 10.3389/fimmu.2023.1200769

**Published:** 2023-05-24

**Authors:** María Botía-Sánchez, Georgina Galicia, Lorena Albaladejo-Marico, Daniel Toro-Domínguez, Maria Morell, Raquel Marcos-Fernández, Abelardo Margolles, Marta E. Alarcón-Riquelme

**Affiliations:** ^1^ GENYO, Center for Genomics and Oncological Research, Pfizer/University of Granada, Andalusian Government, Parque Tecnológico de la Salud, Granada, Spain; ^2^Department of Microbiology and Biochemistry of Dairy Products, Instituto de Productos Lácteos de Asturias (IPLA), Consejo Superior de Investigaciones Científicas (CSIC), Villaviciosa, Asturias, Spain; ^3^Institute for Environmental Medicine, Karolinska Institutet, Stockholm, Sweden

**Keywords:** Lupus, TLR7, intestinal permeability, gut immune cells, microbiota

## Abstract

**Introduction:**

Systemic lupus erythematosus is an autoimmune disease with multisystemic involvement including intestinal inflammation. Lupus-associated intestinal inflammation may alter the mucosal barrier where millions of commensals have a dynamic and selective interaction with the host immune system. Here, we investigated the consequences of the intestinal inflammation in a TLR7-mediated lupus model.

**Methods:**

IgA humoral and cellular response in the gut was measured. The barrier function of the gut epithelial layer was characterised. Also, microbiota composition in the fecal matter was analysed as well as the systemic humoral response to differential commensals.

**Results:**

The lupus-associated intestinal inflammation modifies the IgA^+^ B cell response in the gut-associated lymphoid tissue in association with dysbiosis. Intestinal inflammation alters the tight junction protein distribution in the epithelial barrier, which correlated with increased permeability of the intestinal barrier and changes in the microbiota composition. This permeability resulted in a differential humoral response against intestinal commensals.

**Discussion:**

Lupus development can cause alterations in microbiota composition, allowing specific species to colonize only the lupus gut. Eventually, these alterations and the changes in gut permeability induced by intestinal inflammation could lead to bacterial translocation.

## Introduction

Systemic lupus erythematosus (SLE) is a systemic autoimmune disease characterized by the development of pathogenic antibodies with nuclear antigen reactivity (1). Anti-nuclear antibodies bound to antigens form immunocomplexes (ICs) that deposit on, and damage, multiple tissues (2), including intestinal tissue (3). The etiology of SLE involves both genetic and environmental factors. Among the multiple risk loci identified to increase the likelihood of developing SLE, toll-like receptor 7 (TLR7) gene polymorphisms and copy number variants have been associated with SLE susceptibility (4, 5). Overexpression of TLR7 is known to induce autoimmunity (6) and recently, a *de novo* TLR7 mutation was found to cause a monogenic form of the disease in a young individual (7).

TLR7 is an endosomal receptor expressed in monocytes, macrophages, conventional (cDC) and plasmacytoid (pDC) dendritic cells, and B lymphocytes (8). Both, its overexpression and overactivation (7) have been described to promote SLE progression (9). TLR7 is activated in response to both pathogen and host-derived single stranded RNA (ssRNA) and can drive autoimmune inflammation (10, 11). In B cells, TLR7 acts synergistically with B cell receptor (BCR) activation to promote isotype switching and differentiation into antibody-secreting plasma cells (12). In SLE, the TLR7-signal amplification has been shown to boost autoantibody production, with autoreactive B cells preferentially targeting nuclear antigens (4, 6).

In recent years, compelling evidence has accumulated suggesting that the microbiota is an environmental factor contributing to the pathogenesis of lupus (13–15). Dysbiosis of the intestinal mucosa has been demonstrated in SLE patients and in lupus animal models (16). In the steady state, the intestinal barrier separating the lamina propria from the gut lumen limits the passage of bacteria and regulates the absorption of nutrients, metabolites, ions, and water (17). The selectivity and permeability of the intestinal barrier is largely determined by tight junctions (TJ) formed between adjacent enterocytes. Tight junctions are composed of various claudin molecules and occludin, which connect the inner part of the cells through the cytoskeleton (18, 19). The integrity of the intestinal barrier is also regulated by the interaction between commensals and cells of the intestinal immune system, not only through the immune response of the host but also through the secretion of immunomodulatory metabolites from bacteria (20).

B cells contribute to the intestinal barrier homeostasis by producing large amounts of secretory IgA (sIgA), which is released into the intestinal lumen. IgA binds microorganisms, regulating the composition of the commensal community, intestinal motility, and preventing their translocation into the blood stream (21).

Immune-mediated intestinal diseases and chronic inflammation result in loss of the epithelial barrier integrity because of the inflammatory milieu and the modification of both the commensal community and the metabolic functions they modulate (17, 22, 23). In lupus, disruption of intestinal permeability has been linked to disease exacerbation and translocation of commensals into the system (9, 24). Particularly, in mice with transgenic overexpression of TLR7 (TLR7Tg.1), the overgrowth of *Lactobacillus reuteri* has been associated with a leaky gut, allowing its translocation to extraintestinal tissues worsening the disease (25).

Here, we investigated the intestinal immune response, microbiota composition, and intestinal barrier integrity in a TLR7-mediated lupus model. The TLR7Tg mice carry 8-16 copies of the *Tlr7* gene on the Y chromosome (4). Using this model, we found that lupus-associated intestinal inflammation alters the IgA^+^ B cell response in gut-associated lymphoid tissues, causes intestinal dysbiosis, and alters epithelial barrier tight junction protein expression, which is associated with increased intestinal barrier permeability. The increased gut permeability resulted in a differential systemic humoral response to commensal bacteria and particularly to bacteria that were overrepresented in TLR7Tg mice, likely due to their translocation into the bloodstream.

## Methods

### Mice

C57BL/6J mice were purchased from Charles River. Transgenic mice overexpressing TLR7 (TLR7.Tg) were maintained on a C57Bl/6J genetic background. TLR7.Tg.6 mouse strain herein named TLR7Tg, was obtained from Dr. Darise Farris, Oklahoma Medical Research Foundation, United States (4). According to Boland, et al., in order to generate the transgenic mice overexpressing TLR7 (TLR7.Tg) mice a RP23-139P21 BAC construct including the *Tlr7*, *Tlr8*, and *Tmsb4x* genes, was used. The first 83kb were replaced with a neomycin cassette for allowing the exclusive expression of the *Tlr7* gene. The final construct was injected into C57BL/6-derived zygotes, and 6 positive founders were produced with varying copy number of *Tlr7*. The *TLr7Th.6* strain was a phenocopy of the *yaa* mice having 9-16 copies of the *Tlr7* gene in the Y chromosome resulting in 4-9-fold increase in *Tlr7* mRNA.

All mice were housed and bred in the animal facility at the Biomedical Research Center from the University of Granada under specific pathogen-free (SPF) conditions, provided with a standard chow diet and in a 12h light cycle. Experimental procedures were approved by the Animal Experimentation Committee from the University of Granada and the Spanish Ministry of Agriculture (06/03/2020/035).

### Blood and fecal sample collection

Mice were anesthetized with ketamine at 100mg/kg and xylazine at 5mg/kg in PBS to collect blood through exsanguination *via* cardiac puncture. In addition, blood samples were collected from the saphenous vein at various times during the disease. Blood was allowed to clot overnight at 4°C and then centrifuged at 9560 g for 10 minutes to obtain serum. Serum samples were stored at -20°C until use. Fecal samples were collected under sterile conditions at various times during the disease course. Feces were immediately stored at -20°C until processed.

### Bacterial isolation from feces

Fecal matter was dissolved in PBS (GIBCO) at 100 mg/ml. To remove undigested food, the fecal slurry was centrifuged at 187 g for 10 minutes. The supernatant, containing bacteria, was then centrifuged at 2020 g for 10 minutes. The supernatant, containing free immunoglobulins, was collected and mixed with a protease inhibitor cocktail (Roche) and stored at -20°C until analysis. The pellet, containing bacteria, was washed 2X with PBS 1% BSA for further analysis.

### Detection of immunoglobulins and autoantibodies

To assess anti-dsDNA antibody titers 96-well ELISA plates (ThermoFisher Scientific) were coated with 100µl of protamine sulfate (Sigma) at 500 µg/ml for 45 minutes at 4°C, then calf thymus dsDNA was added (Sigma Aldrich) at 5 µg/ml and the plates were incubated at 37°C for 2 hours followed by overnight incubation at 4°C. Plates were washed 5 times with PBS containing 0.05% Tween 20. Serum samples were added at a dilution of 1:2000 for the quantification of IgG and IgG2c. Samples were incubated at 37°C for 2 hours. After two washes, biotinylated anti-IgG2c (1:1000 in PBST/BSA 1%, Southern Biotech) was added and incubated at 37°C for 30 minutes. After 4X washing, HRP-streptavidin (1:100 in PBST/BSA 1%) was added and incubated for 30 minutes at room temperature. The plates were then washed 4X, 3,3’,5,5’-tetramethylbenzidine (TMB) substrate was added, and the colorimetric reaction was stopped by adding 2N H_2_SO_4_ (Sigma) after 10 minutes. Absorbance was measured at 450 nm and 570 nm using an Infinite200Pro plate reader. Results are represented in optical density (O.D.) values.

Serum anti-sm and anti-RNP antibodies were quantified using the AtheNA Multi-Lyte ANA-III test system (Zeus Scientific A22001) following the manufacturer’s protocol. Serum antibody levels are reported as arbitrary units/milliliter (AU/ml) estimated from a standard curve.

For the inhibition assay, serum samples (diluted 1:1000) were incubated with *B. acidifaciens* (25, 10, 5, 1 x 10^4^, or none) in 200μl for 2 hours at 4 °C. Bacteria were spun down at 1160 g for 5 minutes at 4°C and supernatants were collected to determine by ELISA the IgM anti-dsDNA levels as described above. The O.D. values were used to calculate the percentage of inhibition.

Total IgM, IgA, and IgG2c in serum and feces were measured by sandwich ELISA according to the manufacturer’s instructions (Invitrogen). Briefly, ELISA plates were coated with capture antibodies overnight at 4°C. After washing and blocking, diluted samples (1:100 for IgG2c, 1:5000 for IgA and IgG, and 1:50000 for IgM) were added and incubated for 2 hours at room temperature. The plates were then washed, detection antibodies were added and incubated for 30 minutes. After washing 4 times, TMB substrate was added, and the reaction was stopped with 2N H_2_SO_4_ (Sigma). Absorbance was measured as described above.

To measure specific anti-commensal IgM, IgA, and IgG2c antibodies present in serum, feces from either WT or TLR7Tg mice (0.15 mg) were homogenized in 3ml of PBS, filtered (40 μm) and centrifuged to remove undigested food. The supernatants were collected and centrifuged to spin down the bacteria. The recovered bacteria were heat-inactivated (85°C for 1 hour) and then resuspended in a final volume of 20ml. Isolated fecal bacteria (100 μl/well) were plated on 96-well ELISA plates (ThermoFisher Scientific) and incubated overnight at 4°C. The plates were washed 4X with PBS containing 0.5% Tween 20 (PBST) and serum samples were added at 1:400 dilution in PBST/BSA 0.5% for IgM, 1:20 for IgG2c, and 1:100 for IgA and incubated overnight at 4°C. HRP-anti-IgM (BD) 1:6000, HRP-anti-IgG2c (Southern Biotec) 1:1000, or HRP-anti-IgA (Southern Biotec) 1:6000 were added and incubated for 1.5 hours at room temperature. Plates were then washed, revealed, and measured as described above.

### Flow cytometry

To analyze the B cell response in the small intestine, Peyer’s patches (PP) were excised from the small intestine. The small intestine was emptied, opened longitudinally, and washed with cold HBSS, and cut into 0.5 cm pieces. Epithelial cells were removed by incubating the minced tissue in HBSS with EDTA 3X for 10 minutes at 37°C. The remaining tissue was digested with collagenase D (0.25 mg/ml) and DNAse (0.05 mg/ml) (Roche) at 37°C and at 250 rpm for 30 minutes to obtain a single cell suspension, which was subsequently passed through 100 and 40 µm filters (BD). Peyer’s patches were also digested with collagenase D and DNase and passed through a 40 µm filter (BD) to obtain a single-cell suspension. The isolated cells were stained with live/dead aqua and then incubated with anti-CD16/32. This was followed by incubation with anti-CD19 FITC or BV605, -B220 eF450 or FITC, -CD3/F4/80 PE Cy7, -CD45 APC, -GL7 PE, -CD95 PE-CF594, and -CD138 PercpCy5.5. Cells were then permeabilized (Cytofix/Cytoperm BD) for intracellular staining with anti-IgA PE (Southern Biotec). All antibodies were diluted 1:200 in PBS 1x/EDTA 2 mM/BSA 0.5%

### Flow cytometry analysis of commensal bacteria

To analyze the IgG2c, IgM, or IgA coating of fecal bacteria *ex vivo* by flow cytometry, bacterial fractions isolated from fecal contents were quantified. All bacterial solutions were adjusted to 4x10^9^ bac/ml, then 25 µl (10^8^ bacteria) were plated in 96-well U-bottom plates (ThermoFisher Scientific) and incubated overnight at 4°C with autologous sera diluted 1:2, 1:10, 1:50 or 1:100 in PBS/BSA 1%. After incubation, the bacteria were centrifuged at 1160 X g for 5 minutes at 4°C and washed with PBS/BSA 1%. To avoid unspecific antibody binding, bacteria were blocked with normal rat serum at 20% in PBS/BSA 1% for 15 minutes at 4°C. Bacteria were then washed and stained with biotinylated anti-IgG2a (Southern Biotech, with the ability to specifically recognize IgG2c), anti-IgM APC (eBioscience), or anti-IgA PE (Southern Biotech) all diluted at 1:40 in PBS/BSA 1% for 15 minutes at 4°C. Bacteria incubated with biotinylated-IgG2c were incubated with streptavidin-APC diluted 1:160 in PBS/BSA 1%. Bacteria were then stained with 200 µl of SytoBC (Invitrogen) diluted 1:1000 in PBS/BSA 1% for 15 minutes at 4°C and fixed with PFA 2% in PBS 1x for 30 minutes at 4°C. Samples were acquired on a Canto II cytometer (BD) and analyzed using FlowJo software.

To determine the humoral response against *Bacteroides acidifaciens* by flow cytometry, the protocol was done according to (26) with the following modifications: complement inactivated serum samples were serially diluted and 25 µl were incubated with either 2.5 x 10^5^ *B. acidifaciens* or *P. distasonis* (used as a negative control) for 2h and then washed and centrifuged at 4000rpm. Bacteria were incubated with normal rat serum 20% for 15 minutes at 4°C, followed by incubation with detection antibodies anti-IgM APC Cy7 (Biolegend), IgA PE (Southern Biotech), IgG2c-PE (Southern Biotech), at 1:40 dilution for 15 minutes at 4 C. Bacteria were washed once and stained with 200 µl of SytoBC (1:1000) for 15 minutes at 4°C. After one wash the samples were fixed with 2% PFA for 15 minutes at 4°C, washed and acquired in a Symphony cytometer (BD) and analyzed with FlowJo software. The values of MFI and serum dilution were analyzed to calculate the area under the curve.

### *In vivo* gut permeability assay

Gut permeability *in vivo* was assessed by modifying the protocol described by Zegarra-Ruiz D.F et al. (25). C57BL/6J (B6) and B6.*Tlr7.*6tg mice were weighed and fasted (food and water) for 4 hours, after which the mice were gavaged with fluorescein isothiocyanate (FITC)-coupled dextran 4KDa (Sigma) at 250 mg/kg dissolved in PBS 1x. At this time, water was restored and 3 hours later blood was collected from the saphenous vein. Fluorescence was then measured in serum samples diluted 1:4 in PBS using a plate reader (Infinite 200Pro). The excitation and emission wavelengths were set at 485 and 528 nm, respectively. The autofluorescence emission value of plasma from untreated mice was subtracted from the experimental samples.

### Immunofluorescence

The small intestine was dissected from 32-week-old C57BL/6J (B6) and TLR7Tg mice, and 2 cm segments of distal ileum were obtained, emptied, and opened longitudinally. Ileal segments were fixed in 4% PFA for 30 seconds, then washed in PBS. Peyer´s patches were collected from the ileum and washed in PBS. Ileum and Peyer´s patches tissues were embedded in OCT (Tissue-Tek) and frozen in an isopentane-dry ice mix. Eight-µm sections were hydrated for 20 minutes in TBS and 20 minutes in TBS with Tween20 0.05% (TBS-T). Tissue sections were then blocked with TBS-T/BSA 5%, 2 mg/ml of anti-CD16/CD32, and 10% normal rat and mouse serum for 30 minutes at room temperature. Slides were briefly washed in TBS-T and incubated with anti-EpCam APC at a 1:500 dilution (Invitrogen) and anti-Claudin 1 Alexa Fluor 488 at 1:50 dilution (Invitrogen) or anti-Occludin Alexa Fluor 594 at 1:100 dilution (Invitrogen) overnight at 4°C. Cell nuclei were stained with Hoechst 33342 (1µM, Sigma-Aldrich) for 5 minutes at room temperature. Slides were mounted with SlowFade Diamond Antifade Medium (Thermo Fisher Scientific, Waltham, MA) and images of tissue sections were captured using either a ZEISS Axiocam 506 mono 5X/0.15 and 10X/0.45 objectives or a Zeiss 710 Laser Scanning Microscope, a Zeiss Plan-Apochromat 63X/1.40 NA oil-immersion DIC M27 objective (aperture pinhole= 1.0 Airy Unit), a Zeiss Plan-Apochromat 10X/0.45 or 20X/0.8 NA objective and the Zeiss ZEN 2010 software. Fluorescence was acquired sequentially using different lasers for excitation and different photomultipliers for the detection of all fluorescence signals.

### Bacterial strains and growth conditions

*Bacteroides acidifaciens* DSM15896 and *Parabacteroides distasonis* DSM20701 were obtained from the Leibniz Institute DSMZ-German Collection of Microorganisms and Cell Cultures GmbH. Culture medium for *Bacteroides acidifaciens* DSM15896 was 50% (v/v) of Brain-Heart Infusion (BHI, Oxoid Ltd) and 50% (v/v) of Reinforced Clostridial Medium (RCM, Oxoid Ltd)-supplemented with 5% (v/v) heat-inactivated fetal bovine serum (Sigma). *Parabacteroides distasonis* DSM20701 was cultured in Gifu Anaerobic Medium (GAM; HiMedia Laboratories). Both bacteria were first grown on the surface of agar plates at 37°C in an MG500 anaerobic chamber

(Don Whitley Scientific; atmosphere of 10% (v/v) H_2_, 10% CO_2_, and 80% N_2_) for 48 hours. Subsequently, isolated colonies were inoculated in broth media and incubated O/N, and these pre-cultures were used as fresh inoculum for the preparation of 30ml broth cultures. After O/N growth, cells were washed with PBS and pellets were resuspended in 1 mL of the corresponding medium supplemented with 20% trehalose, Bacterial stocks were preserved at -80°C until used. The identity of the bacterial stocks was corroborated by partially sequencing the 16S rRNA gene using the primers 27F 5’-AGAGTTTGATCCTGGCTCAG-3’ and 1492R 5’- GGTTACCTTGTTACGACTT-3’) (27, 28).

### Microbial DNA isolation and 16S rRNA gene sequencing

Microbial DNA was extracted from fecal samples using the QIAamp^®^ Fast DNA Stool Mini Kit “Pathogen Detection” (QIAcube/QIAGEN). Quality and quantity were assessed by NanoDrop (ThermoFisher). Amplicon libraries for the 16S rRNA gene hypervariable region (V4) were generated using universal 16S PCR Forward Primer = 5’TCGTCGGCAGCGTCAGATGTGTATAAGAGACAGCCTACGGGNGGCWGCAG3´ and Reverse Primer = 5’GTCTCGTGGGCTCGGAGATGTGTATAAGAGACAGGACTACHVGGGTATCTAATCC. The resulting amplifications were analyzed by electrophoresis in a 2% agarose gel. Amplified products were then quantified by Qubit (ThermoFisher), pooled using 50 ng/sample, and filtered in a column (Omega Bio-Tek) and using DNA AMPure XP magnetic beads (Beckman Coulter). Quality control was performed by High Sensitivity Bioanalyzer (Agilent) in order to check that there were no remaining primers being the library size of about 400 bp, as expected. The resulting purified pool was diluted to 4 nM, quantified again by Qubit, and sequenced using the MiSeq Reagent Kit v3 in a MiSeq System according to the Illumina protocols.

### 16S data preprocessing, OTU assignment, and diversity analyses

Quality control was performed on raw sequencing reads (fastq format) using FastQC. The end-50 pairs of bases of reads were cut and removed. All preprocessing and statistical analyses were performed with qiime2 version-2021.8. Denoising was performed using the dada2 algorithm in order to better discriminate between true sequence diversity and sequencing errors. Chimeric sequences were identified and removed using the uchime algorithm. Operational taxonomic units (OTUs) were identified and annotated using sklearn and the greengenes training input files provided by qiime. Unaligned sequences were filtered, and a phylogenetic tree was constructed using the raxml algorithm. Alpha diversity between groups of mice was estimated by the Kruskal-Wallis test obtaining Plelou-evenness and Faith_PD values. Beta diversity was estimated using Bray-Curtis dissimilarity and UniFrac distance. Differential abundance analysis between groups of mice was performed by ancom (analysis of the composition of microbiomes with bias correction). Finally, LEfSe (linear discriminant analysis effect size) was performed to measure the effect size of each taxon for each group of mice and the significance level was assessed by the Kruskal-Wallis test.

### Statistical analysis

Statistical analysis was done with GraphPad Prism V9.5.0 software (GraphPad La Jolla, CA) and R. Significant differences between two groups were calculated using a two-tailed Mann–Whitney test. P values were considered significant if they were equal to or less than 0.05.

## Results

### TLR7-mediated lupus alters intestinal permeability

To determine disease kinetics in TLR7Tg mice, the levels of serum autoantibodies and the splenomegaly were measured at different time points. TLR7Tg animals showed increasingly elevated levels of anti-dsDNA IgG2c compared to controls from 12 weeks of age (O.D. WT 0.157 ± 0.053 vs 0.373 ± 0. TLR7Tg) to 32 weeks of age (O.D. 0.116 ± 0.025 WT vs 0.401 ± 0.100 TLR7Tg) (**Supplementary Figure 1A**). Levels of anti-sm and anti-ANAs antibodies were also elevated at 32-weeks of age (**Supplementary Figures 1B, C**), confirming disease development. Splenomegaly also developed progressively in TLR7Tg mice, with the weight of the spleen being 2-fold greater in 12-week-old TLR7Tg mice than in WT animals and 8-fold greater in TLR7Tg mice than in controls at 32 weeks of age (**Supplementary Figure 1D**). Accordingly, the spleen/body weight ratio was significantly reduced in 32 weeks old TLR7Tg mice compared to WT mice (**Supplementary Figure 1E**).

**Figure 1:**
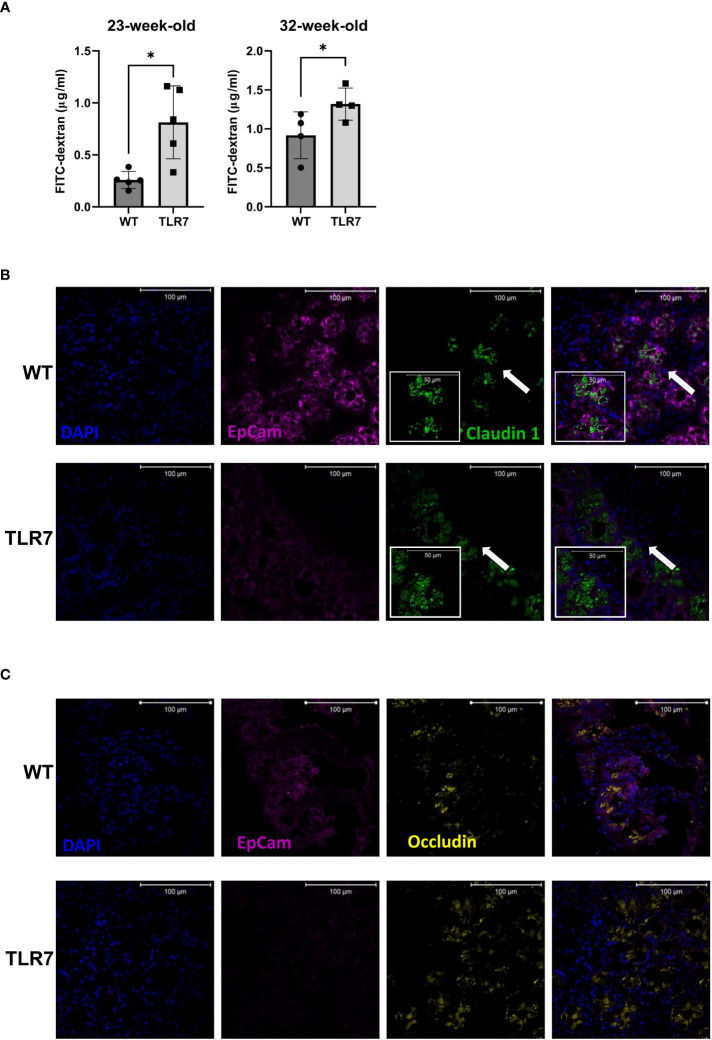
Increased gut permeability was accompanied by altered patterns of occludin and claudin 1. **(A)** Gut permeability was measured *in vivo* with FITC-dextran at two different time points. Representative data from three independent experiments with n=4-5 mice/group. Mean with SD. Representative staining of **(B)** claudin 1 and **(C)** occludin in a histological transversal section of the distal ileum from WT control mice and TLR7Tg mice with established lupus. **(B)** The insert (arrow) shows a digital zoom (2.0) selected from the image. All images were captured using a Zeiss Plan-Apochromat 63X/1.40 NA oil-immersion DIC M27 objective (aperture pinhole= 1.0 Airy Unit). A: unpaired Mann-Whitney test. *p ≤ 0.05.

We then analyzed whether disease alterations in the immune response have any impact on the gut associated immune response and the epithelial barrier homeostasis. To address this issue, we used fluorescein isothiocyanate (FITC)-dextran to test the paracellular permeability *in vivo* in TLR7Tg and WT mice at 23 and 32 weeks of age. TLR7Tg mice exhibited significantly increased gut permeability at both timepoints compared to age- and sex-matched WT mice, with significant differences in the systemic dextran concentration that passed through the intestinal barrier (**Figure 1A**).

To further investigate the changes in intestinal permeability, we used immunofluorescence to analyze the expression of the tight junction proteins claudin-1 and occludin, proteins that ultimately determine the structure and function of the barrier (29). Cytoplasmic dense expression of claudin-1 with a vesicle-like pattern was observed in the ileal epithelium of 32 weeks old TLR7Tg mice (**Figure 1B**). This expression pattern differed from that in the ileal epithelium of control mice, where claudin-1 was expressed adjacent to the membrane epithelial cell adhesion molecule 1 (Ep-CAM1) (**Figure 1B**). Similarly, occludin changed its expression pattern in the ileal epithelium of TLR7Tg mice with established disease compared to control mice (**Figure 1C**). The WT ileal epithelial expression of occludin was characterized by a homogeneous pattern that changed to a cytoplasmic vesicle-like pattern in TLR7Tg diseased mice. Altogether, these results demonstrate that TLR7 overexpression induces epithelial barrier dysmorphism, as evidenced by changes in the expression patterns of the tight junction proteins, possibly due to an ongoing inflammatory process.

### Lupus inflammation affects the gut immune response and supports an increased local IgA production

**Figure 2:**
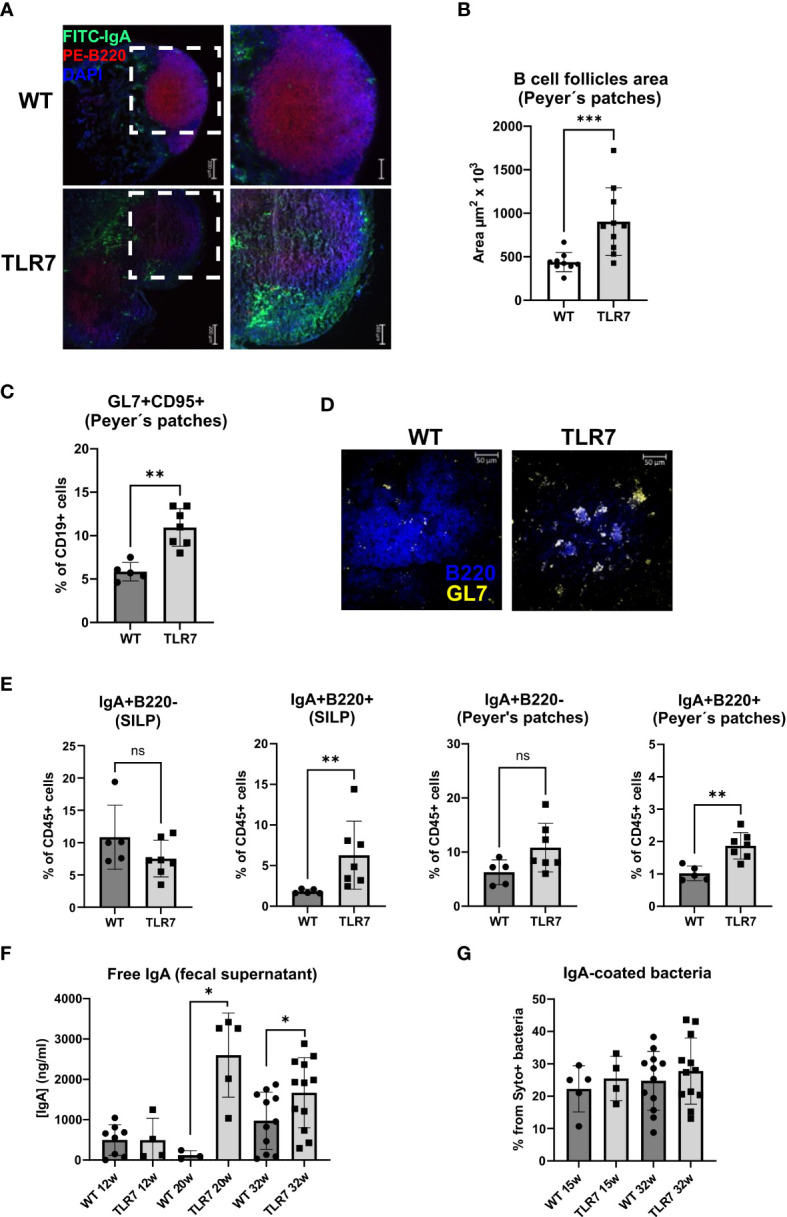
Mice with lupus display disrupted Peyer's patches and changes in the IgA response. **(A)** Peyer's patches representative staining with IgA-green, B220-red, and DAPI-blue. **(B)** Mean ± SD of the area of B cell follicles in the Peyer's patches from 32 week-old TLR7Tg mice (n=10) and controls (n=9). **(C)** Frequency of GC B cells determined by flow cytometry (WT n=5; TLR7 n=7), the experiment was replicated 2 times with similar results. **(D)** Representative staining GL7-yellow and B220-blue of Peyer’s patches from TLR7Tg and WT mice (n= 5 mice per group). **(E)** Mean of the percentages of CD45^+^ IgA^+^ B220^+^ or B220^-^ cells in the Peyer’s patches and SILP (WT n=5; TLR7 n=7). Representative data of three independent experiments with similar results. **(F)** Concentration of free IgA in feces from TLR7Tg mice and WT controls at different time points of lupus progress measured by ELISA (representative data from three independent experiments with n= 3-12 mice/group). **(G)** Mean of the frequency of IgA-coated fecal bacteria determined by flow cytometry (representative data from four independent experiments with n=6 mice/group). Images in A were captured using a ZEISS Axiocam 506 mono 5X/0.15 and 10X/0.45 objectives. **(A, D–F)** Graphs represent mean ± SD. Unpaired Mann-Whitney test. ns (non significant): p>0.05; *p ≤ 0.05; **p ≤ 0.01; ***p ≤ 0.001.

We then investigated whether systemic inflammation that occurs in TLR7Tg mice also involves the intestinal immune response by determining the activation of B cells in the gut-associated lymphoid tissue (GALT). Histologically, we observed that the organized structure of Peyer´s patches was lost in the TLR7Tg mice. While in WT Peyer´s patches the B cell zone had a regular circular and compacted shape, in the TLR7Tg Peyer´s patches the B cell zone was divided into two enlarged and dispersed areas with an irregular, disrupted, amorphous shape (**Figure 2A**). Quantification of the B-cell follicle areas in the Peyer´s patches proved a statistically significant enlargement of these areas in TLR7Tg mice compared to WT mice (**Figure 2B**). Then, germinal center (GC) B cells were assessed by flow cytometry and immunofluorescence in the Peyer´s patches. TLR7Tg mice showed an increased frequency of CD95^+^GL7^+^ B cells as compared to WT animals (**Figure 2C**), mirroring the increased frequency of GC B cells in the spleen (**Supplementary Figure 2A**). Furthermore, in Peyer´s patches tissue sections, TLR7Tg B220^+^ B cells showed increased expression of GL7 (**Figure 2D**) as compared to WT Peyer´s patches. The upregulation of GL7 on B cells in Peyer’s patches of TLR7Tg mice indicates increased B cell activation.

Because IgA is the most abundantly secreted isotype in the normal intestinal mucosa we analyzed the IgA gut-associated immune response in 32 weeks old mice with lupus. IgA-producing cells found in the small intestine lamina propria (SILP), and PP were characterized. The frequency of SILP IgA^+^ plasma cells (B220^-^ IgA^+^) was similar in TLR7Tg and WT mice, whereas these cells were slightly, but not significantly increased in the PP of TLR7Tg mice compared to WT controls (**Figure 2D**). The frequency of IgA^+^ B cells (B220^+^ IgA^+^) was significantly higher in both SILP and PP of TLR7Tg mice when compared with WT mice (**Figure 2E**). Histological analysis of PP showed an increased presence of IgA^+^ cells in and around the B cell zone in TLR7Tg mice (**Figure 2A**), confirming what we had observed by flow cytometry.

To analyze whether this increase in newly generated IgA^+^ B cells correlates with an alteration in the secretory IgA response against commensal bacteria, we measured the IgA coating of fecal bacteria by flow cytometry and the amount of free IgA in fecal samples by ELISA. TLR7Tg 12-week-old mice showed no differences in fecal-free IgA levels compared to WT controls, while IgA levels were significantly increased in 20 weeks old TLR7Tg mice compared to controls (**Figure 2F**). Thirtytwo weeks old TLR7Tg mice had higher concentration of free IgA in their feces than did their age-matched WT controls. Interestingly, the levels of free IgA were maintained between 20 and 32 weeks (**Figure 2F**). Intriguingly, WT and TLR7Tg mice had similar levels of IgA-coated fecal bacteria at 15, 20, and 32 weeks of age (**Figure 2G**). Thus, the increased frequency of IgA^+^ class-switched B cells in the SILP and Peyer’s patches contributed to higher levels of free IgA in the feces but did not alter the total percentage of IgA-coated bacteria.

### Gut dysbiosis is associated with TLR7-mediated lupus

**Figure 3:**
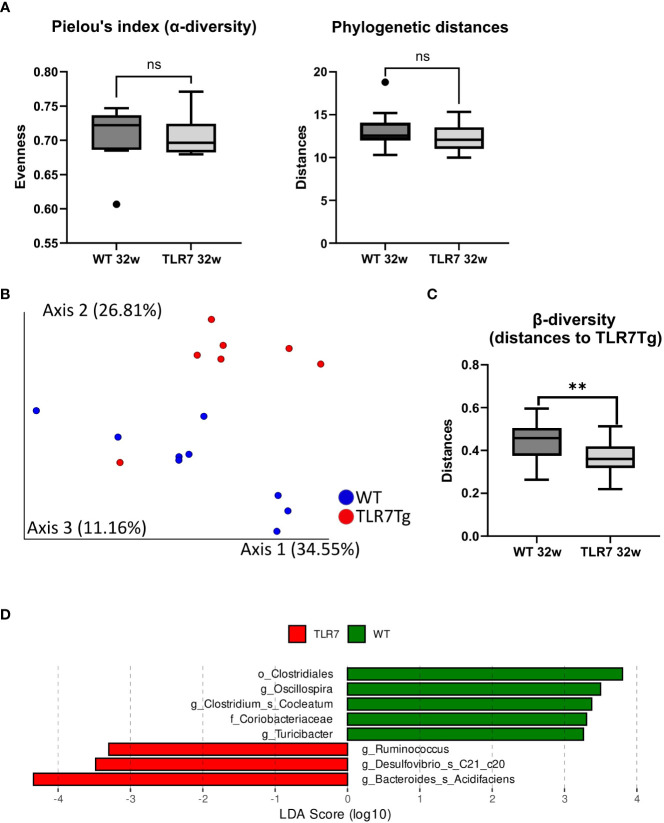
TLR7Tg mice show gut microbiome dysbiosis. **(A)** Analysis of the alpha diversity measured by Pielou’s index and phylogenetic distances; Faith_PD test. **(B)** Pairwise permanova analysis and **(C)** Bray-Curtis dissimilarity analysis of microbiome beta diversity between WT and TLR7Tg mice with established lupus. **(D)** Linear discriminant analysis effect size (LDA) scores of altered taxa in TLR7Tg mice compared with WT mice. **(A, B)** Graphs represent mean with SD. Unpaired Mann-Whitney test. ns (non significant): p>0.05; **p ≤ 0.01.

The changes in the intestinal IgA^+^ response led us to hypothesize that the microbiota composition might be different between the two mouse genotypes since IgA controls the microbiota composition of the gut mucosa (30). To investigate how changes in the intestinal-IgA response shape the microbiota composition in the TLR7Tg mice, we collected fecal samples from mice in the early and established phase of the disease and performed 16s rRNA sequencing. Alpha diversity (**Supplementary Table 1**) was similar between TLR7Tg mice and WT controls, and so were the phylogenetic distances (**Figure 3A**). However, principal component analysis (PCA) revealed differences in the gut microbiota composition between TLR7Tg mice and WT mice (**Figure 3B**) which was supported by a permanova analysis of the beta diversity (**Figure 3C**). Although the overall species richness was similar between the two genotype-associated microbiomes, we observed species that differentially colonized the gut of TLR7Tg mice compared to control mice. Among them we found *Bacteroides acidifaciens*, *Desulfobivrio c20_c21*, and the genus *Ruminococcus* (**Figure 3D**). On the other hand, the genera *Oscillospira* and *Clostridium*, along with the family *Coriobacteriaceae* appeared only in the intestine of control mice (**Figure 3D**). The Analysis of Compositions of Microbiomes (ANCOM) confirmed the differential abundance of *B. acidifaciens*, which was enriched only in TLR7Tg mice in the early (data not shown) and established phases of the disease (**Supplementary Figures 3A, B**). These results demonstrated a dysbiosis in the intestinal mucosa of the TLR7Tg mice.

### Alterations in gut permeability induced variations in the humoral systemic response against commensal bacteria

**Figure 4:**
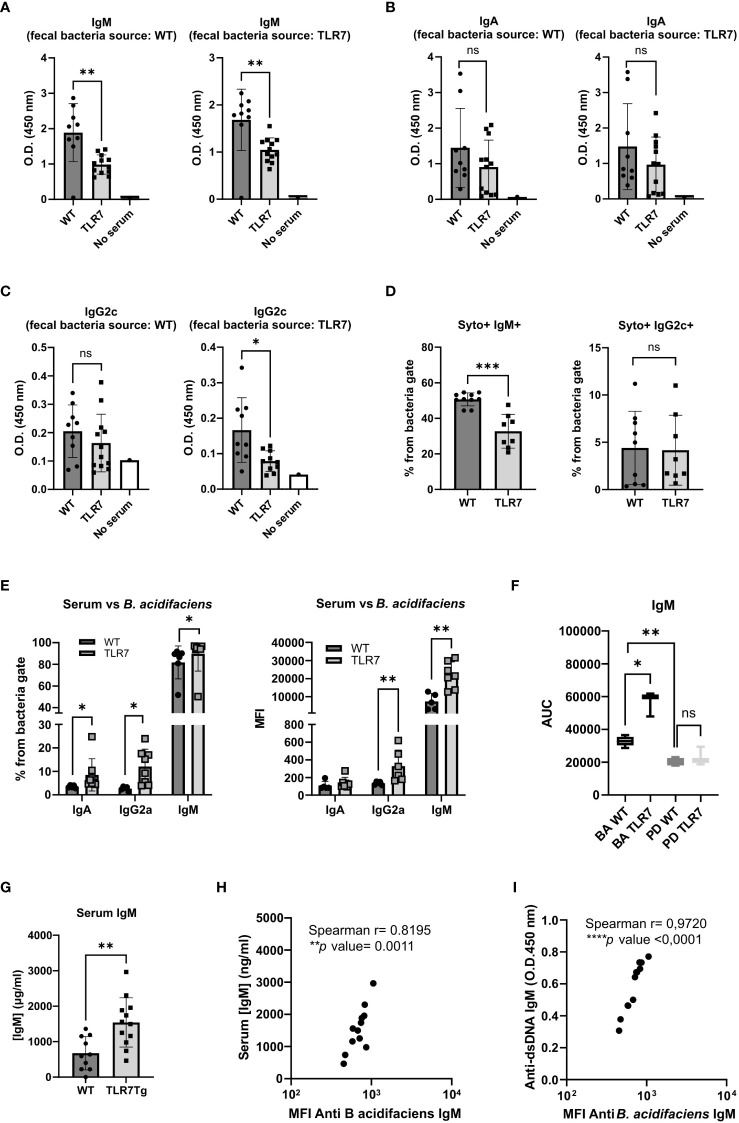
Changes in the humoral immune response against autologous and heterologous fecal commensal bacteria by TLR7Tg mice. Mean levels of **(A)** IgM, **(B)** IgA, and **(C)** IgG2c vs autologous and heterologous fecal bacteria as determined by ELISA. Serum and feces collected from TLR7Tg at 32-weeks of age. (WT n=9; TLR7Tg n=12). **(D)** Proportion of bacteria recognized by serum IgM and IgG2c determined by flow cytometry (WT n=10; TLR7Tg n=8). **(E)** The serum antibody response against *B. acidifaciens* as assessed by flow cytometry (WT n=6; TLR7Tg n=8). **(F)** Serum IgM response against *B. acidifaciens* and *P. distasonis.* Serial dilutions of sera of WT or TLR7Tg were incubated with purified bacteria and the response was assessed by Flow Cytometry. The area under the curve obtained by the alignment of MFI at each dilution was calculated for each sample (WT n=5-9; TLR7Tg n=3-12). Data represents results from 2 separate experiments. Data is presented as boxes and whiskers from min to max. **(G)** Total IgM in serum determined by ELISA (WT n=15, TLR7Tg n=12. **(H)** Correlation between total IgM in serum and specific levels of IgM reactive against *B. acidifaciens*. **(I)** Correlation of anti-dsDNA IgM antibodies and levels of anti-*B. acidifaciens* IgM antibodies. **(A–E)** and **(G)** Data represent mean with SD. Unpaired Mann-Whitney test. **(H)** Spearman correlation. ns (non significant): p>0.05; *p ≤ 0.05; **p ≤ 0.01; ***p ≤ 0.001; ****p ≤ 0.0001.

Disruptions of gut barrier integrity are often associated with bacterial translocation to the mesenteric lymph nodes and the liver, which in turn can induce a systemic antibody responses to commensal bacteria (31). To assess whether fecal bacteria induce a humoral response beyond the intestinal tissue in TLR7Tg mice, we measured the levels of serum IgM, IgA, and IgG2c reactive to gut bacteria in TLR7Tg mice with established lupus and in WT control mice. Sera were tested with autologous and heterologous fecal commensals from age-matched mice (WT sera *vs*. WT commensals or WT sera *vs.* TLR7Tg commensals and vice versa). The serum IgM response to commensal microbiota was significantly reduced in TLR7Tg when compared to WT mice regardless of the source of the fecal bacteria (**Figure 4A**). Serum IgA anti-commensal response to autologous and heterologous commensals was unchanged in WT and TLR7Tg mice (**Figure 4B**). Interestingly, the IgG2c response to WT microbiota was comparable between WT and TLR7Tg mice, however the IgG2c levels were significantly reduced in TLR7Tg mice when tested against their autologous commensal bacteria compared to the WT IgG2c response to the TLR7Tg commensals (**Figure 4C**).

In light of the differential response of serum IgG2c to autologous and heterologous commensals, we used flow cytometry to compare head-to-head only the autologous response of WT and TLR7Tg serum antibodies to their own fecal microbiota (**Supplementary Figure 4**). We confirmed that TLR7Tg mice had significantly lower IgM levels to their own microbiota when compared to WT mice (**Figure 4D**), whereas serum levels of IgG2c against their autologous commensal bacteria were not different between WT and TLR7Tg (**Figure 4D**). These data suggest that TLR7-overexpression mainly alters the serum IgM response to commensal bacteria.

Given the differential serum antibody response to total gut commensals in TLR7Tg mice, we further investigated the serum antibody response to the species with the most significant overabundance in the feces of TLR7Tg mice, *B. acidifaciens* (**Supplementary Figures 3A, B**). The proportion of *B. acidifaciens* coated by serum IgA, IgG2c, and IgM antibodies from TRL7Tg mice was significantly higher compared to controls (**Figure 4E**). However, while the proportion of IgA- and IgG2c-coated *B. acidifaciens* did not exceed 20% of bacteria population, the frequency of IgM-coated *B. acidifaciens* was over 80% in TLR7Tg and control mice. Similar results were observed when the antibody levels were analyzed by MFI (**Figure 4E**). Thus, we reasoned that, given that WT mice did not have intestinal *B.acidifaciens*, the Ig serum response may correspond to a natural polyclonal response while in the TLR7Tg mice it may correspond to an adaptive and specific IgM response. To determine this, sera from WT and TLR7Tg mice were serially diluted and incubated with either *B. acidfiaciens* or another unrelated bacterial species (*P. distasonis*) as negative control, not present in neither WT nor TLR7Tg mice. The IgM response level was calculated as the area under the curve of MFI values of serum dilutions, as described by Danska et al. (26). We found that the IgM response to *P. distasonis* was equally negligible in WT and TLR7Tg (**Figure 4F**) and that the WT IgM response to *B. acidifaciens* was higher than the IgM response to *P. distasonis* (**Figure 4F**). On the contrary, the antibody response to *B. acidifaciens* was significantly higher in sera from TLR7Tg mice compared with WT mice (**Figure 4F**; **Supplementary Figure 5A**), hence the IgM serum antibodies from TLR7Tg mice specifically recognized *B. acidifaciens*.

Moreover, the total amount of serum IgA (**Supplementary Figure 5B**), IgG2c (**Supplementary Figure 5B**), and IgM (**Figure 4G**) were increased in TLR7Tg mice. For IgM we found a significant direct correlation of total IgM serum levels and the anti-*B. acidifaciens* IgM serum levels. This correlation was not found in WT controls (**Supplementary Figure 5C**), indicating the presence of a specific response to the bacteria in TLR7Tg mice (**Figure 4H**). Moreover, serum levels of IgM anti-*B.acidifaciens* directly correlated with serum levels of IgM anti-dsDNA antibodies (**Figure 4I**). Furthermore, incubation of serum with *B. acidifaciens* inhibited the binding of IgM antibodies to dsDNA (**Supplementary Figure 5D**). Taken together, these results indicate a possible antigenic cross-reaction between antibodies to *B. acidifaciens* with antibodies to anti-dsDNA.

## Discussion

In a TLR7-mediated lupus model, we investigated the consequences of the intestinal epithelial barrier disruption throughout the course of disease on the systemic humoral immune response to commensals. Here we showed that the development of autoimmunity was accompanied by structural and functional changes in the gut B-cell response, gut microbiome dysbiosis showing the overabundance of *B. acidifaciens*, and a differential peripheral immune response against these bacteria.

The GCs of Peyer´s patches are the site of B cell expansion and differentiation for antibody secretion in response to commensals (32, 33), however in autoimmunity, Peyer´s patches may instead amplify the autoimmune response (34). In the TLR7Tg mice the Peyer´s patches were enlarged and unstructured and showed extensive GC formation with an increased frequency of GC B cells (GL7^+^ CD95^+^ CD19^+^) and IgA^+^ B cells (B220^+^ IgA^+^). Similarly, the SILP of lupus mice, showed an accumulation of IgA^+^ B cells, which may have either migrated from the Peyer’s patches or been generated in the isolated lymphoid follicles (ILFs) in the lamina propria (35). The increased frequency of IgA^+^ B cells was associated with greater IgA secretion into the intestinal lumen. Increased levels of fecal-free IgA levels were detected in 20-week-old TLR7Tg mice and persisted for up to 32 weeks. This increase occurred concomitantly with the increase in serum levels of IgG anti-dsDNA antibodies. On the other hand, fecal free IgA antibodies were tested against nuclear antigens, but we did not detect autoantigen recognition (data not shown), contrary to what has been observed previously in other lupus models (36).

Although there were increased levels of free intestinal IgA, the frequency of IgA-coated fecal bacteria was unaltered in TLR7Tg mice. This phenomenon has also been observed in an autoimmune arthritis model (K/BxN mice), where IgA levels increased but the frequency of IgA-coated bacteria did not (37). IgA secreted in the gut lumen plays a crucial role in controlling the gut microbiota composition. Any change in specificity, affinity, or quantity may modify the gut microbiota ecology (30, 38). We found an overabundance of *B. acidifaciens* in the feces of TLR7Tg mice. *B. acidifaciens* has also been reported to be one of the species with increased abundance in TLR7.1Tg female mice (25), which indicates that *B. acidifaciens* is associated with the lupus phenotype regardless of sex and animal facility conditions.

*B. acidifaciens* has been reported to play various beneficial roles in different pathologies, such as metabolic pathologies by modulating insulin resistance and energy metabolism (39), non-alcoholic liver disease by preventing hepatocyte apoptosis (40), and pathogen colonization resistance by producing propionate (41). *B. acidifaciens* has also been shown to promote IgA production in the colon and Peyer’s patches (39, 42). However, it can also play a detrimental role in uremic toxicity induced by antibiotics and chemotherapy by producing indole sulfate (43). Similarly, *B. acidifaciens* negatively contributes to gut barrier disruption by inducing a decrease in the expression of zonulin-1 and claudin-1 (44). Thus, depending on the context, *B. acidifaciens* can exert divergent roles. In contrast, the family *Coriobacteriaceae* was enriched only in WT mice. This family belongs to the class Actinobacteria, which has been associated with low risk of developing lupus (45). Oral transfer of *B. acidifaciens* will help us gain more insight into the role of this bacterial species in lupus pathogenesis.

The epithelial barrier controls the paracellular transport of solutes and small molecules and prevents the transport of proteins, lipids, and microbial-derived molecules (46). Intestinal inflammation and dysbiosis alter the integrity of the epithelial barrier by modifying the expression of adhesion molecules, production of mucus, and antimicrobial molecules (47). These changes result in increased gut barrier permeability, commonly known as “leaky gut”. Autoimmune diseases such as inflammatory bowel disease, rheumatoid arthritis, type 1 diabetes, and lupus have been associated with leaky gut (48). Intestinal epithelial cells are attached to their neighbors by a continuous belt of tight junctions in the most apical side of the basolateral membrane. The integrity and proper function of the epithelial barrier depends on the appropriate expression of tight junction proteins, such as occludin and claudin-1. We did not observe differences in expression of the tight junction proteins, instead, we observed that TLR7Tg mice had a redistribution of the transmembrane proteins occludin and claudin-1 away from the cell membrane tight junctions. Both proteins were localized into cytoplasmic vesicle-like structures. This type of redistribution of tight junction proteins upon inflammatory cytokines exposure has been associated with discontinuity in the epithelial membrane due to microtubule retraction (49).

Based on previous reports (50) indicating that a persistent leaky gut may lead to excessive bacterial translocation, we reasoned that the increased intestinal permeability in TLR7Tg mice could trigger an altered humoral response against gut commensals. Such analysis revealed that serum IgM antibody levels to commensals (autologous and heterologous) were significantly decreased in TLR7Tg mice. This is in line with a previous report demonstrating decreased circulating levels of IgM antibodies against bacterial endotoxin core (EndoCAb) in lupus patients (51) which was suggested to be associated with greater exposure to commensals in lupus patients due to leaky gut. Similarly, in lupus patients, IgM anti-phosphorylcholine (PC) antibody levels, which cross-react with bacterial polysaccharides, have been reported to diminish when disease activity increases (52). Hence, in TLR7Tg mice the reduction in anti-commensal IgM antibodies could be related to both a high disease activity and increased exposure to microbial components.

Increased serum levels of IgA, IgG2c, and IgM antibodies against *B. acidifaciens* that were found in TLR7Tg mice, compared with WT controls, can be taken as an indicator of gut bacteria translocation to the system. A similar increased systemic IgG response was associated with translocation of gut bacteria healthy mice (31). It has also been demonstrated that gut microbiota induces a homeostatic humoral response, not only restricted to IgG, but also IgA and IgM. Therefore, we determined all three classes (53) against the most abundant bacteria in the gut of TLR7Tg mice. Although we found an increased adaptive IgG2c and IgA response in TLR7Tg mice against *B. acidifaciens*, the IgM response was unexpectedly higher. Those higher levels of IgM anti-*B.acidifaciens* antibodies directly correlated with total serum IgM. This suggests that higher total IgM may be the result of continued B cell activation (34) where part of the anti-*B. acidifaciens* response, as compared to the *P. distasonis* response, is not polyclonal, but specific and differentially increased in the TLR7Tg lupus male mice, while that of *P. distasonis* is comparably low in both strains. Furthermore, the highly significant correlation of IgM anti-*B. acidifaciens* antibody levels with IgM anti-dsDNA and the inhibition of binding of serum IgM to dsDNA by *B. acidifaciens* indicate that IgM antibodies produced in TLR7Tg mice might have a cross-reaction between bacterial and self-antigens. Altogether, these results suggest that the specific humoral response against *B. acidifaciens* can detrimentally contribute to disease development.

## Conclusion

We conclude that increased gut permeability, the overactive immune responses, both local and systemic, and gut dysbiosis can modify each other, creating a vicious feed-forward loop. Within this relationship, TLR7-mediated lupus inflammation affects the gut-associated B cell response, the epithelial barrier integrity, and the systemic humoral response to commensals, altering the immune response that may fuel autoimmunity.

## Data availability statement

The original contributions presented in the study are publicly available. This data can be found here: https://www.ebi.ac.uk/ena/browser/view/PRJEB61233.

## Ethics statement

The animal study was reviewed and approved by Animal Experimentation Committee from the University of Granada and the Spanish Ministry of Agriculture (06/03/2020/035).

## Author contributions

MB-S and GG carried out all experiments. RM and AM did bacterial strains cultures. LA-M helped with anti-dsDNA ELISAs and flow cytometry bacterial staining. MM helped with the breeding of mice and with anti-sm and ANAs ELISAs. DT-D did all bioinformatic analyses of microbiota sequencing. GG and MA-R supervised all analyses and suggested new approaches. MB-S and GG wrote the original manuscript and MA-R thoroughly revised the document. All authors contributed to the article and approved the submitted version.
