# Supplementary material for: Gut epithelial barrier dysfunction in lupus triggers a differential humoral response against gut commensals
Source: Front Immunol. 2023 May 24;14:1200769. doi: 10.3389/fimmu.2023.1200769 (PMC10280985; doi:10.3389/fimmu.2023.1200769)
Supplement: Supplementary file 1 [file DataSheet_1.pdf]

Gut epithelial barrier dysfunction in lupus triggers a differential humoral response against commensals

SUPPLEMENTARY MATERIAL

Supp. Table 1.

	Group 1	Group 2	H	p-value	q-value
**Faith_ PD**	TLR7 (n=7)	WT (n=9)	0.22	0.6338	0.6338
**Evenness**	TLR7 (n=7)	WT (n=9)	0.3389	0.5604	0.5604

Table 1. Pairwise comparison of alpha diversity by Kurskal-Wallis test.

Supp. Figure 1.

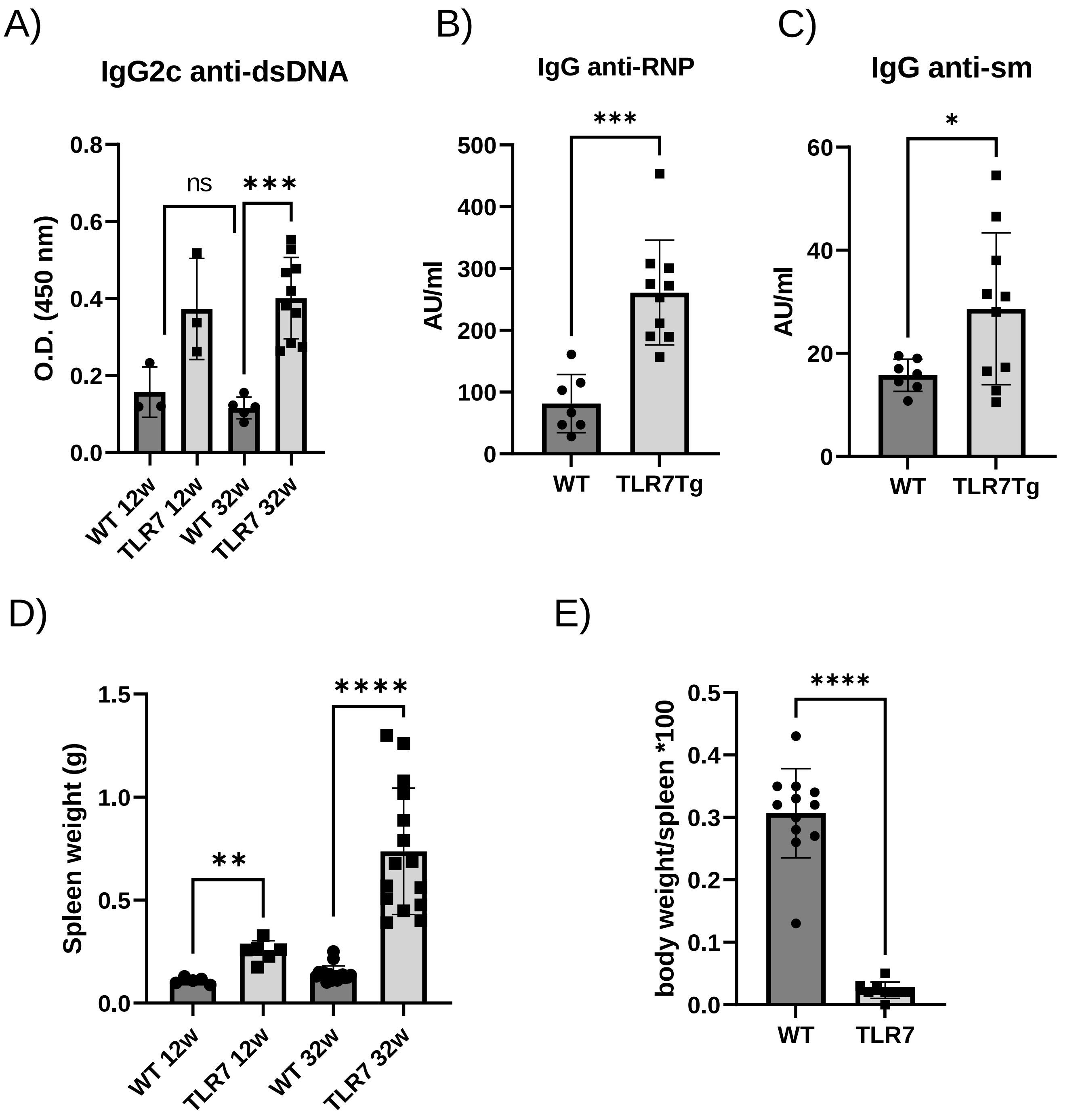

Supp. Figure 1. Overexpression of TLR7 induces the generation of autoantibodies and splenomegaly. A) anti-dsDNA IgG2c at 12- and 32-weeks, B) anti-RNP IgG and C) anti-sm IgG at 32-weeks in serum from TLR7Tg and WT mice. D) Weight of spleen from 12- and 32-week-old mice. E) ratio of body weight/spleen of 32-week-old TLR7Tg and WT mice. 3-10 mice were used from each genotype and age. Data represent three independent experiments with similar results. Error bars indicate SD and the stars above represent p values. A-C: Data represent mean with SD. Unpaired Mann-Whitney test. **$p \leq 0.01$; ***$p \leq 0.001$;****$p \leq 0.0001$.

Supp. Figure 2.

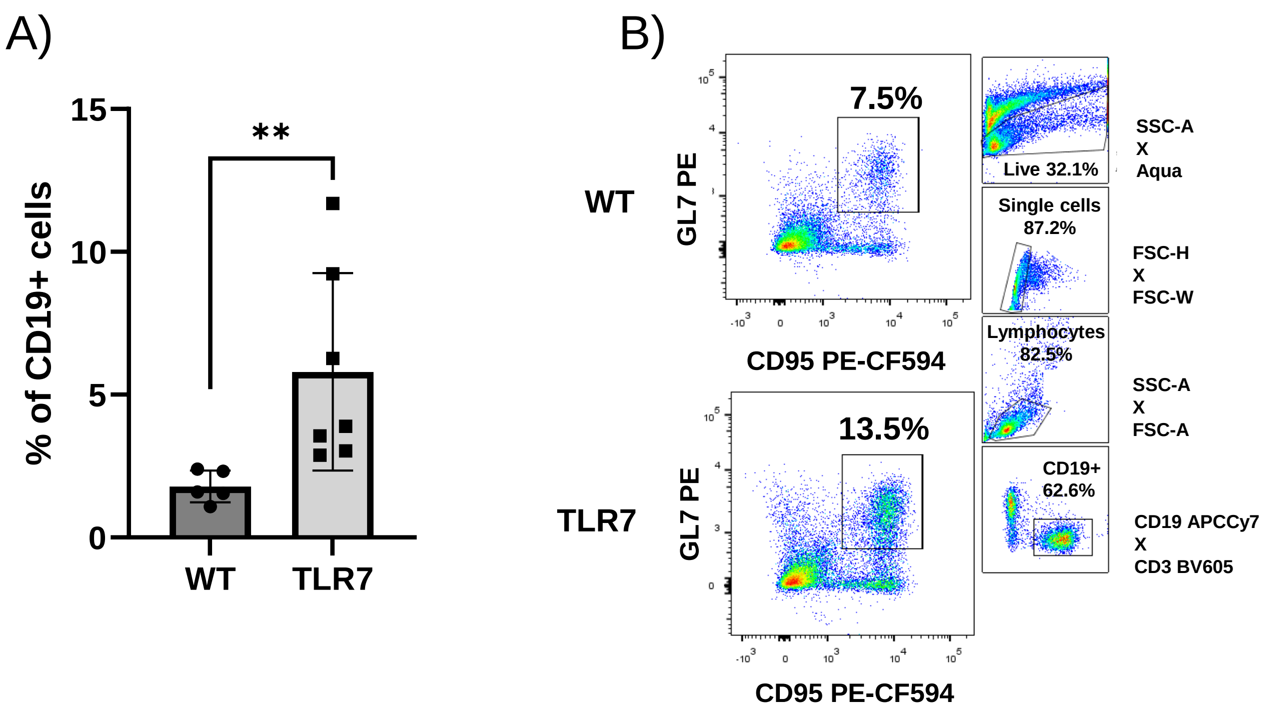

Supp. figure 2. Germinal centers B cells in the spleen and gating strategy for GC B cell detection. A) Mean with SD of the proportion of germinal center B cells in the spleen of TLR7Tg mice with established lupus and WT controls. Analysis of GC was done by flow cytometry (WT n=5; TLR7Tg n=7) B) Gating strategy to determine GC B cells in the Peyer's patches. Unpaired Mann-Whitney test. **p≤0.01.

Supp. Figure 3.

A)

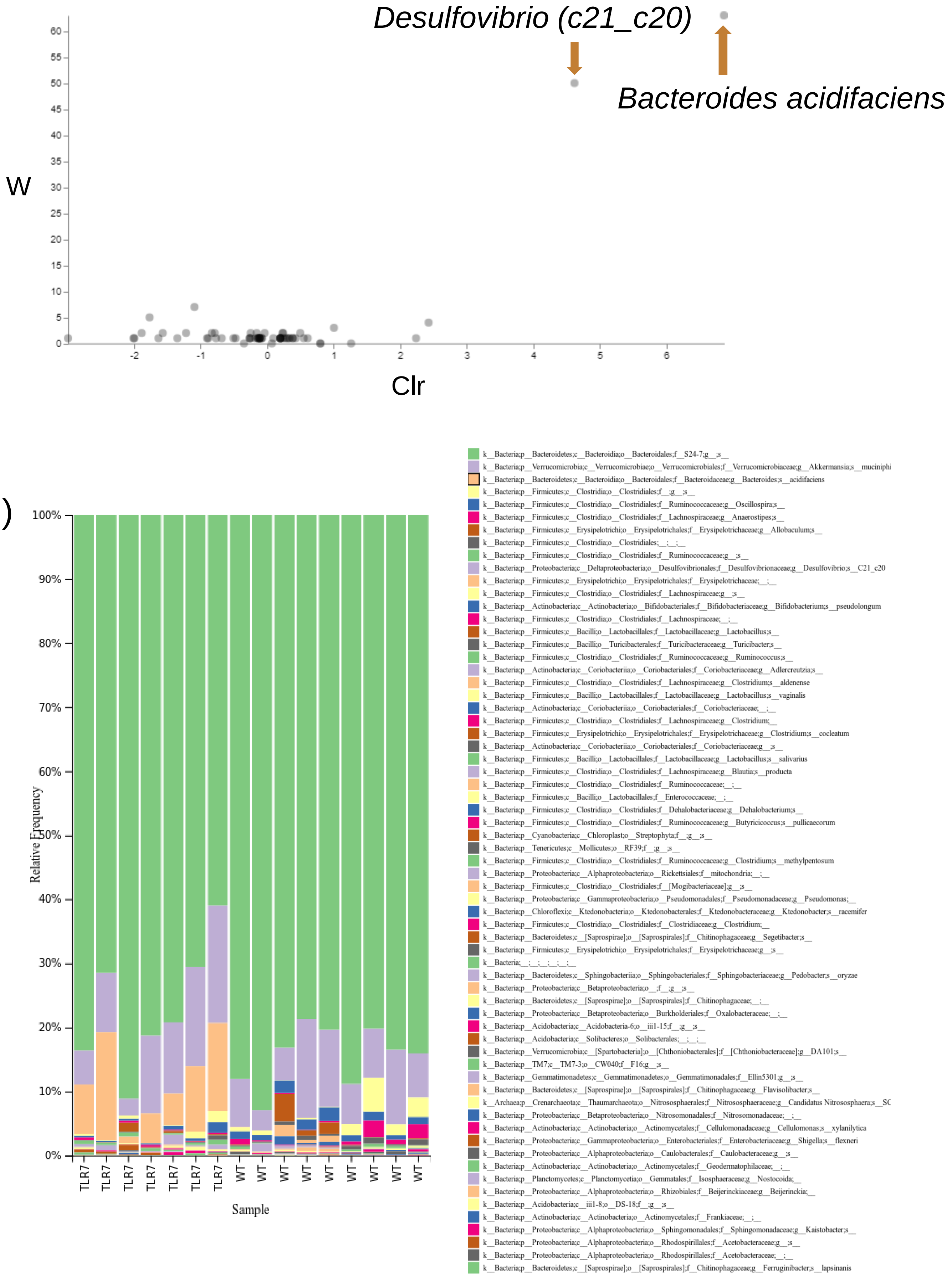

Supp. Figure 3. Increased abundance of *Bacteroides acidifaciens*. A) Analysis of the differential abundance of taxons found in TLR7Tg mice with lupus and WT mice by an analysis of compositions of microbiomes (ANCOM). B) Bar chart showing the abundance of the noted species in fecal samples from TLR7Tg mice with established lupus and WT age-matched mice (WT n=9; TLR7Tg n=7).

Supp Figure 4.

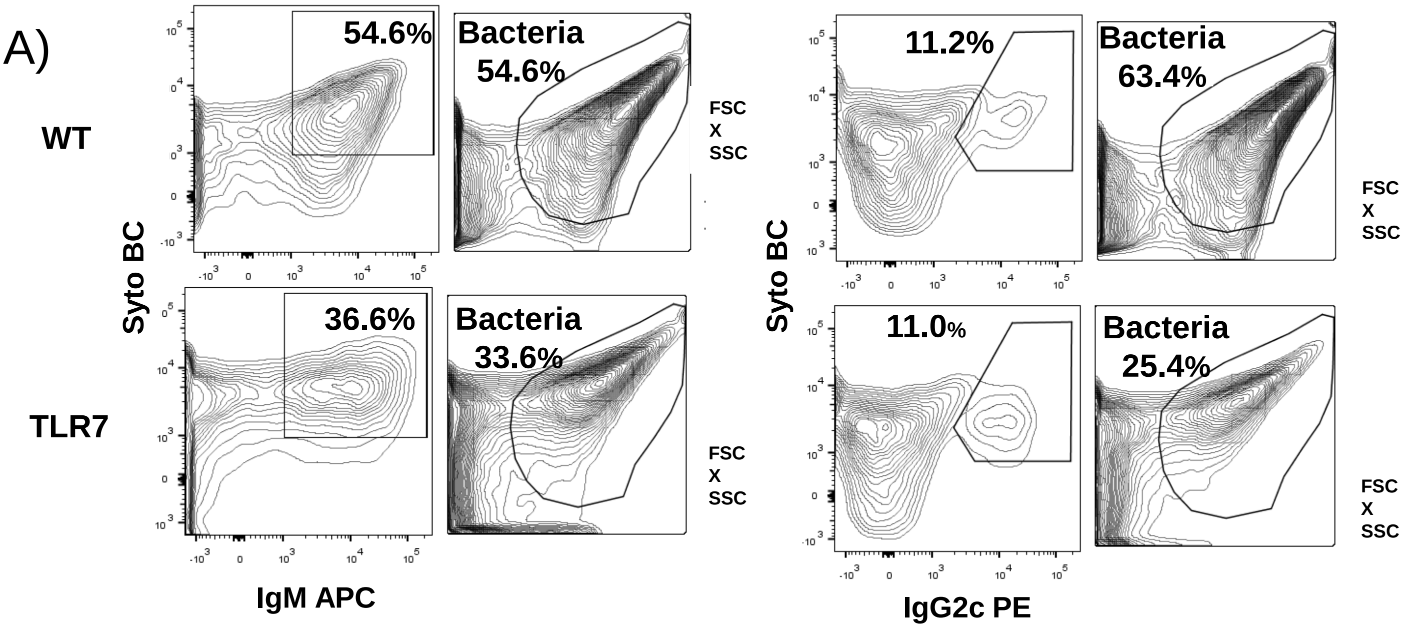

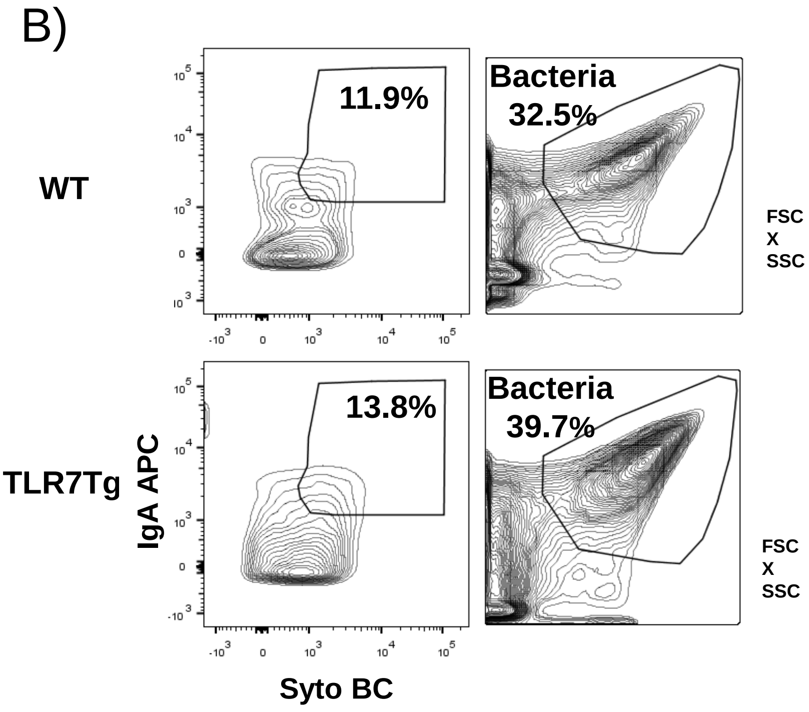

Supp. Figure 4. Gating strategy to analyze commensal bacteria. A) Representative plots of serum IgM and IgG2c binding to autologous fecal bacteria as measured by flow cytometry. B) Flow cytometry representative plots of fecal bacteria coated by IgA.

Supp. Figure 5.

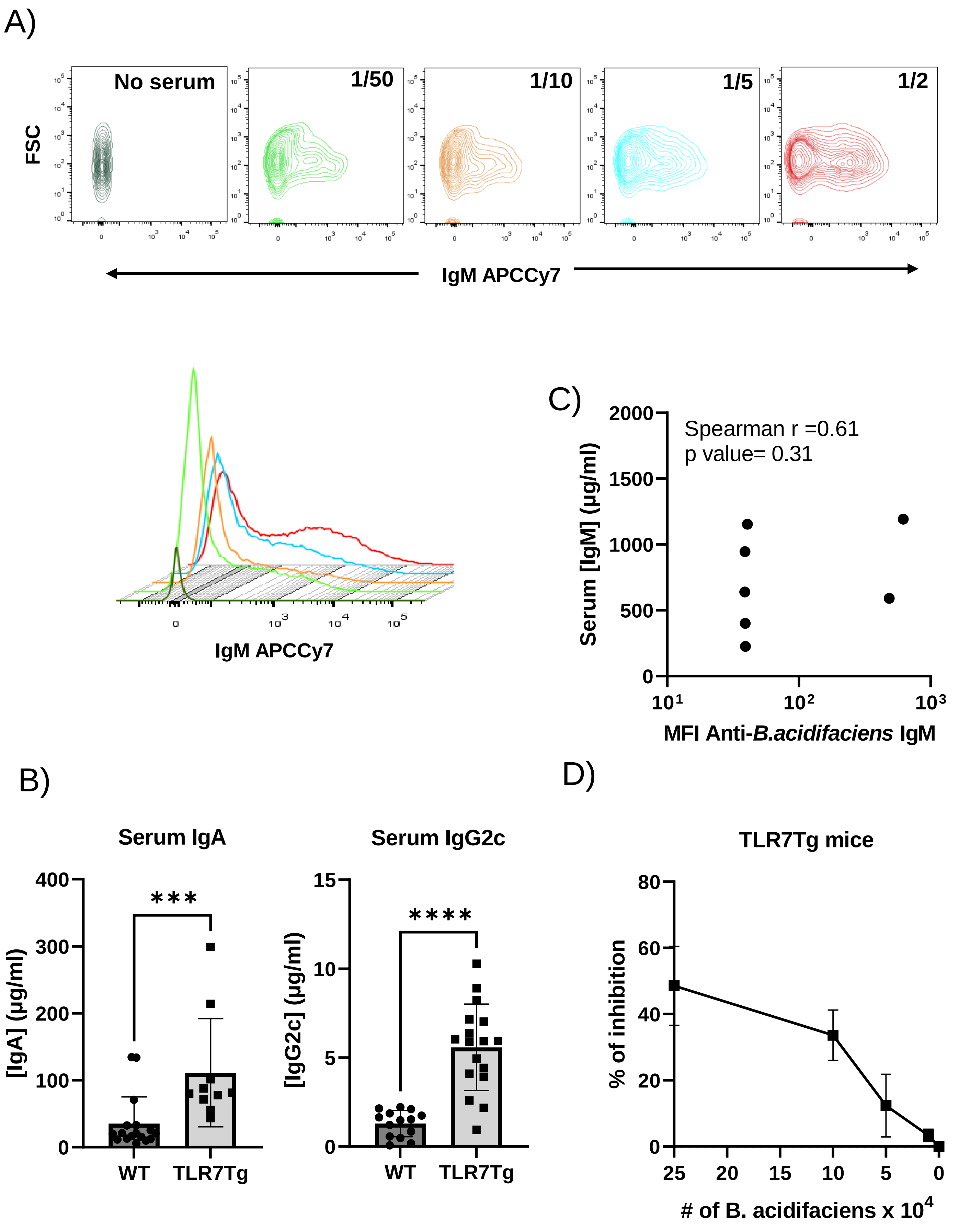

Supp. Figure 5. Humoral response against *B. acidifaciens*. A) Representative plots of IgM^{+} *B. acidifaciens* fraction at each serum dilution tested and comparison of the MFI at each condition. B) IgA and IgG2c in serum of WT and TLR7Tg as determined by ELISA (WT n=15, TLR7Tg n=12). C) Correlation between total IgM in serum and specific levels of IgM reactive against *B. acidifaciens* in WT mice. D) Percentage of inhibition of binding of IgM antibodies to dsDNA by B. acidifaciens in TLR7Tg mice (n=6). B: Data represent mean with SD. Unpaired Mann-Whitney test. H: Spearman correlation. ****p$\leq$0.0001.
